# Multidetector CT findings of primary pleural angiosarcoma : a systematic review, an additional cases report

**DOI:** 10.1186/s40644-021-00435-1

**Published:** 2022-01-12

**Authors:** Bo Mi Gil, Myung Hee Chung, Ki-Nam Lee, Jung Im Jung, Won Jong Yoo, Soon Seog Kwon, Heejeong Lee

**Affiliations:** 1 Department of Radiology, Bucheon St. Mary’s Hospital, College of Medicine, The Catholic University of Korea, 327, Sosa-ro, Bucheon-si, Gyeonggi-do 14647 Republic of Korea; 2 Department of Radiology, Dong-A University Hospital, Busan, Republic of Korea; 3 Kosin University Gospel Hospital, Busan, South Korea; 4 Department of Radiology, Seoul St. Mary’s Hospital, College of Medicine, The Catholic University of Korea, Seoul, Republic of Korea; 5 Division of Allergy and Pulmonary, Department of Internal Medicine, BUCHEON St. Mary’s Hospital, College of Medicine, The Catholic University of Korea, Seoul, Republic of Korea; 6 Department of Pathology, Bucheon St. Mary’s Hospital, Colloge of Medicine, The Catholic University of Korea, Seoul, Republic of Korea

**Keywords:** Primary Pleural Angiosarcoma, Pleural origin, Immunohistochemical studies

## Abstract

**Background:**

To demonstrate and analyze the relatively common imaging findings in this rare primary pleural angiosarcoma (PPA).

**Case presentation:**

Three cases of PPA, proven by video-assisted thoracic surgery biopsies are retrospectively reviewed. Patients were all male. Age ranges from 65 to 75 years old age (mean; 69). Major chief complaints were dyspnea and chest pain. One has a history of colon cancer, the other has a tuberculosis history and the other has no known history. Multidetector chest CT and PET CT were all done. Immunohistochemical studies were performed including CD31, CD34, or factor VIII-related antigen, vimentin, and cytokeratin. We also review the literatures on recently published PPA. All masses were from 1 to 10 cm. All three patients had multiple pleural based masses, which were ovoid in shape with relatively sharp margin in unilateral hemithorax. Multiple small circumscribed pleural masses are limited in the pleural space in two patients, whereas two, huge lobulated masses about up to 10 cm were present with pleural and extrapleural involvement in one patient. In two patients with pleural mass only, multiple pleural masses were only seen in parietal pleura in one patient and were in both visceral and parietal pleura in one patient. Pleural effusion were found in one side in one patient and in both sides in one patient. One angiosarcoma was arised from chronic tuberculotic pleurisy sequelae. All pleural masses are heterogenous with irregular internal low densities in all patients. Hematogenous metastases were found in liver, vertebra, rib in one patient, and were in lungs with mediastinal lymph node metastases in the other patient. Three patients survived for longer than 3months after diagnosis, but continued to deteriorate rapidly. Two patients underwent chemotherapy after surgical excision, and the other one with multiple metastases treated chemotherapy after CT-guided biopsy, but eventually all died. As a result of comparative analysis of a total of 13 patients’ images including 10 cases previously published, there was pleural effusion in all except 2 cases.

**Conclusions:**

PPA were all necrotic without any vascularized enhancing nature, and manifested as unilateral circumscribed or localized pleural-based masses.

**Supplementary Information:**

The online version contains supplementary material available at 10.1186/s40644-021-00435-1.

## Background

Angiosarcoma is a rare malignant tumor that originates in the endothelial cells of blood vessels within the skin, soft tissue, breast, liver, spleen, and other organs. This disease accounts for only 1 – 2% of the total soft tissue sarcomas. According to previous radiologic reports, thoracic angiosarcoma can be classified into lung, mediastinum, pleura, and chest wall origins, depending on where it occurs (1). Lung metastases from extrathoracic angiosarcomas have been described and are common in the natural history of angiosarcoma in the thorax. Primary pleural angiosarcoma (PPA) was first reported in 1943, which is an extremely rare malignancy [1]. PPA can be defined as extrapulmonary angiosarcoma involving the pleura and extrapleural regions. Markers suggestive of epithelial (skin, etc.) and pulmonary origins should be negative.

Until recently, only about 19 PPA patients had been reported in the English literature (3). All PPA cases to date were reported by pathologists, oncologists or cardiothoracic surgeons. The radiologic findings in the PPA cases known to date were nonspecific. We analyzed our three cases more details in the radiologic images and compared them with previously reported cases.

## Materials and methods

We retrospectively reviewed chest multidetector computed Tomography (MDCT) and positron emission tomography-computed tomography (PET CT) images for the most recent three years in three patients diagnosed with PPA. All three patients were male and the mean age was 69 years (range; 65 – 75years). Their chief complaints were dyspnea, chest pain, and cough for about one month. Three had no history of occupational disease, including asbestosis exposure. One of the three was treated for tuberculosis (TB) 40 years ago and later developed TB chronic empyema. Another patient was diagnosed with synchronous cancer (colon cancer) by colonoscopy performed at the time of the visit (Table 1). All three had no angiosarcoma in intra- and extra-thoracic areas other than the pleura on the preoperative examination.

**Table 1:** Clinical data of three cases of primary pleural angiosarcoma

Age/Sex	65/M	75/M	67/M
Chief complain	dyspnea	left chest pain & weakness	dyspnea and cough
Cancer history	None	None	Synchronous colon cancer
Occupational history	None	None	None
TB history	None	Old TB pleurisy	None
Survival (months)	5	1	4

*TB* Tuberculosis

These patients were finally identified with PPA using immunohistochemical studies, including CD31, CD34, factor VIII-related antigen, vimentin, S-100, and cytokeratin, in tissues obtained through video-assisted-thoracic-surgery (VATS) biopsies. According to the pathology papers written by Hart et al. (4), epithelioid angiosarcoma (EA) consists of sheets, nodules, and trabeculae of infiltrative epithelioid to spindled cells with intensely eosinophilic cytoplasm, and shows strongly positive reactivity for factor VIII, CD31, Fli-1, and vimentin and almost negative reactivity for S100. However, the tissues are mainly positive for cytokeratin (CK), but can variably display CD34 and epithelial membrane antigen (EMA) positivity. We set the criteria for differentiation based on the degree of cytologic atypia and mitoses, the focal areas of vessel formation, and a sheeted growth pattern comprising most of the malignancy to differentiate epithelioid hemangioendothelioma from epithelioid angiosarcoma [2].

All enhanced CT images were obtained during full inspiration in the supine position using a MDCT scanner with 64 or more channels. The detailed CT parameters were tube voltage, 120 kVp; tube current standard dosage (reference mAs, 60 – 120) with automatic exposure control; and slice thickness, 1.5 – 2 mm. The CT images were evaluated with both lung (width, 1500 HU; level, -700 HU) and mediastinal (width, 450 HU; level, 60 HU) window settings. Each image was analyzed by two thoracic radiologists with 27 and 5 years of experience, respectively, as faculty chest radiologists.

In the analysis of the published article, only patients with chest plain and/or CT images were used. The image results were based on the paper descriptions, as correlated with the relevant figures. Thus, the characteristics were focused on the image findings of the 10 patients collected.

## Case presentation

This study was approved by the Institutional Review Board of the three hospitals and informed consent was not required.

Our cases were diagnosed as epithelioid type PPA by pathologic examination. All immunochemical reactions except for cytokeratin were strongly positive. Table 2 shows a summary of the imaging findings of our recently discovered patients. All masses were from 1 to 10 cm. All three patients had multiple pleural-based masses, which were ovoid with relatively sharp margins in unilateral hemithorax. One was on the right side and two were on the left side. Multiple small circumscribed pleural masses were limited to the pleural space in two patients, whereas two, huge biconvex masses about up to 15 cm were present with pleural and extra-pleural involvement in one patient. In two patients with pleural masses only, multiple pleural masses were seen in the parietal pleura in one patient and in both the visceral and parietal pleura in one patient. Pleural effusion was found on one side in one patient and on both sides in one patient. One angiosarcoma developed as a sequela of chronic tuberculous pleurisy without pleural effusion. All pleural masses were heterogeneous with irregular internal low densities in all patients. Hematogenous metastases were found in the liver, vertebra, and rib in one patient, and in the lungs with mediastinal lymph node metastases in the other patient. All three patients survived for longer than three months after diagnosis but continued to deteriorate rapidly.

**Table 2:** Summary of multidetector chest CT findings in our three cases

Image findings	Patient 1 (65/M)	Patient 2 (75/M)	Patient 3 (67/M)
Pleural effusion	Yes	No	Yes
Bilateral or unilateral	Bilateral		Unilateral
High-density effusion (hemothorax)	No		No (4000 cc on pleurodesis)
Pleural nodule			
Single or multiple	Multiple	Multiple	Multiple
Mass size	1-2 cm	6 cm, 8.4 cm	2.8 cm~10cm
Mass shape	Oval, discrete	Oval, biconvex	Irregular, conglomerated
Mass location	Parietal pleura	Pleura and extrapleural	Visceral and parietal pleura
Mass net-enhancement	Poorly enhancing	Poorly enhancing (10 HU)	Moderate enhancing (18.6 HU)
Homogeneity	Heterogeneous	Heterogeneous	Heterogeneous
Internal calcification/ air	No	Calcification	No
Internal necrosis	Yes	Yes	Yes
Directly invasion of adjacent structures	No	Chest wall, Lt. 6-8^th^ ribs	Liver invasion
Distant metastasis	No	Lung, ribs, vertebrae, lymph nodes	Adrenal gland, scapula, ribs, vertebrae, and sacrum

*CT* computed tomography; *HU* hounsfield unit

**Fig. 1:**
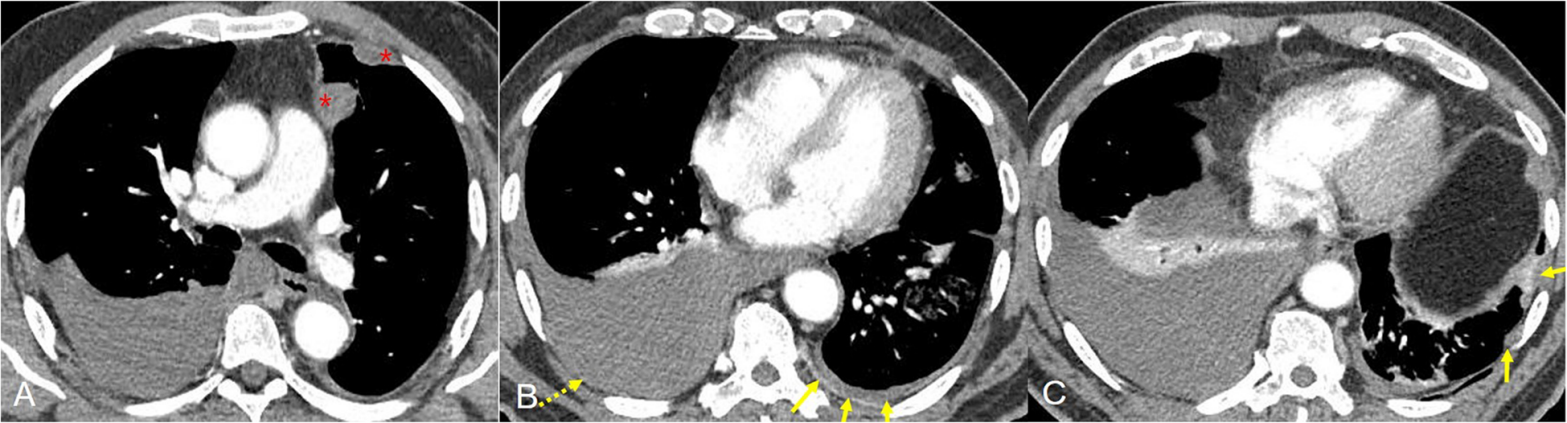
A 65-year-old man diagnosed with primary pleural angiosarcoma. Enhanced, multidetector chest CT scans showed multiple, small nodules of poorly enhancing nature in the left parietal pleura(asterisk) (**A**). There were also right large pleural effusion and left small pleural effusion with thin, parietal pleural enhancement (**B**, **C**)

First, a 65-year-old male presented with dyspnea without known underlying disease or specific history. There was a large amount of bilateral pleural effusion (R > L) on the chest plain and chest CT, taken at the time of admission. Additionally, the chest CT showed multiple, 1 – 2 cm-sized pleural-based nodules, just in the left parietal pleural sites and smooth, thin parietal pleural enhancement on contrast-enhanced CT. Bloody pleural effusion drainage was performed and malignant cells were identified on cytologic examination (Fig. 1). PET-CT showed mild fluorodeoxyglucose (FDG) uptake in the pleural nodules and no extrathoracic malignancy or metastasis. Pleural nodule biopsy and right lower lobar wedge resection were performed by VATS. Pathologically, the left pleural nodule was diagnosed as an endothelial cell malignant tumor. In immunohistochemical staining, CD31, Ki-67 (50%), CD32, CD34, factor VIII-related antigen, and vimentin were strongly positive, but calretinin, desmin, EMA, and CK5/6 were negative. This suggests a probable endothelial cell malignancy. Finally, angiosarcoma of an epithelioid lymphangioendothelial lineage cell type was diagnosed, so chemotherapy (gemcitabine monotherapy plus paclitaxel) was initiated.

**Fig. 2:**
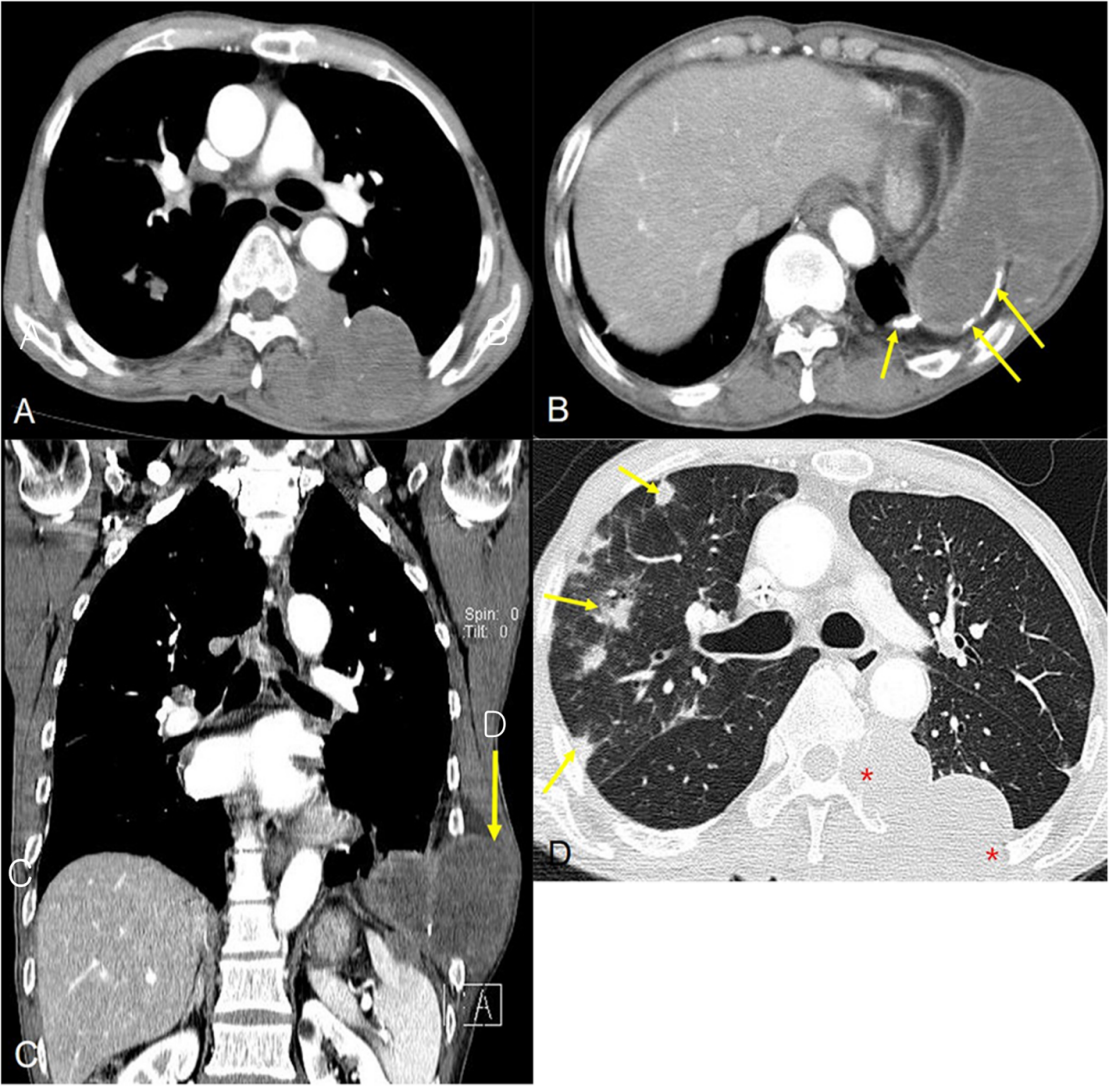
Primary pleural angiosarcoma in a 75-year-old man with a history of tuberculosis. Enhanced multidetector CT scan (**A**, **B**) showed two, sharply demarcated, biconvex, hypodense masses, arising from chronic tuberculous empyema sequelae with calcific plaques. These masses were directly invaded the extrathoracic regions, resulting in adjacent left 6-8^th^ ribs destruction (**B**, **C**). Lung scan (**D**) CT showed multiple hematogenous metastases in both lung fields, and left rib posterior arc change

The second patient was a 75-year-old male with left chest pain and weakness. The patient had a history of anti-tuberculous therapy 40 years ago and left chronic pleurisy sequela on chest PA. The contrast-enhanced chest CT showed two, separated, conglomerated pleural and extrapleural masses about 6 and 8.4 cm each (Fig. 2). These bulky chest wall masses were hypodense and slightly enhanced on each septated margin with direct rib destruction (left 6^th^, 7^th^, and 8^th^ ribs). One mass seemed to have arisen from preexisting pleural calcific plaques. There were accompanying distant metastases (multiple lung metastatic nodules, right 4^th^ rib metastases, and axial vertebral bodies). Also, there were multiple metastatic lymph nodes in both lower paratracheal, subcarinal, both hilar, and left interlobar areas. CT-guided lung biopsy using 17 gage(G) coaxial cutting needle was performed to obtain the tissues to fix in the formalin. Specimens by CT guided biopy was mostly suitable for large extrapleural mass. In immunohistochemical staining, CD31, CD34, factor VIII-related antigen, and vimentin were strongly positive, but calretinin and CK5/6 were negative. Under an angiosarcoma diagnosis, although chemotherapy (gemcitabine monotherapy plus Antazax and Cisplan was performed, there was no improvement in the patient’s condition. After one month, he was discharged with a poor prognosis.

**Fig. 3:**
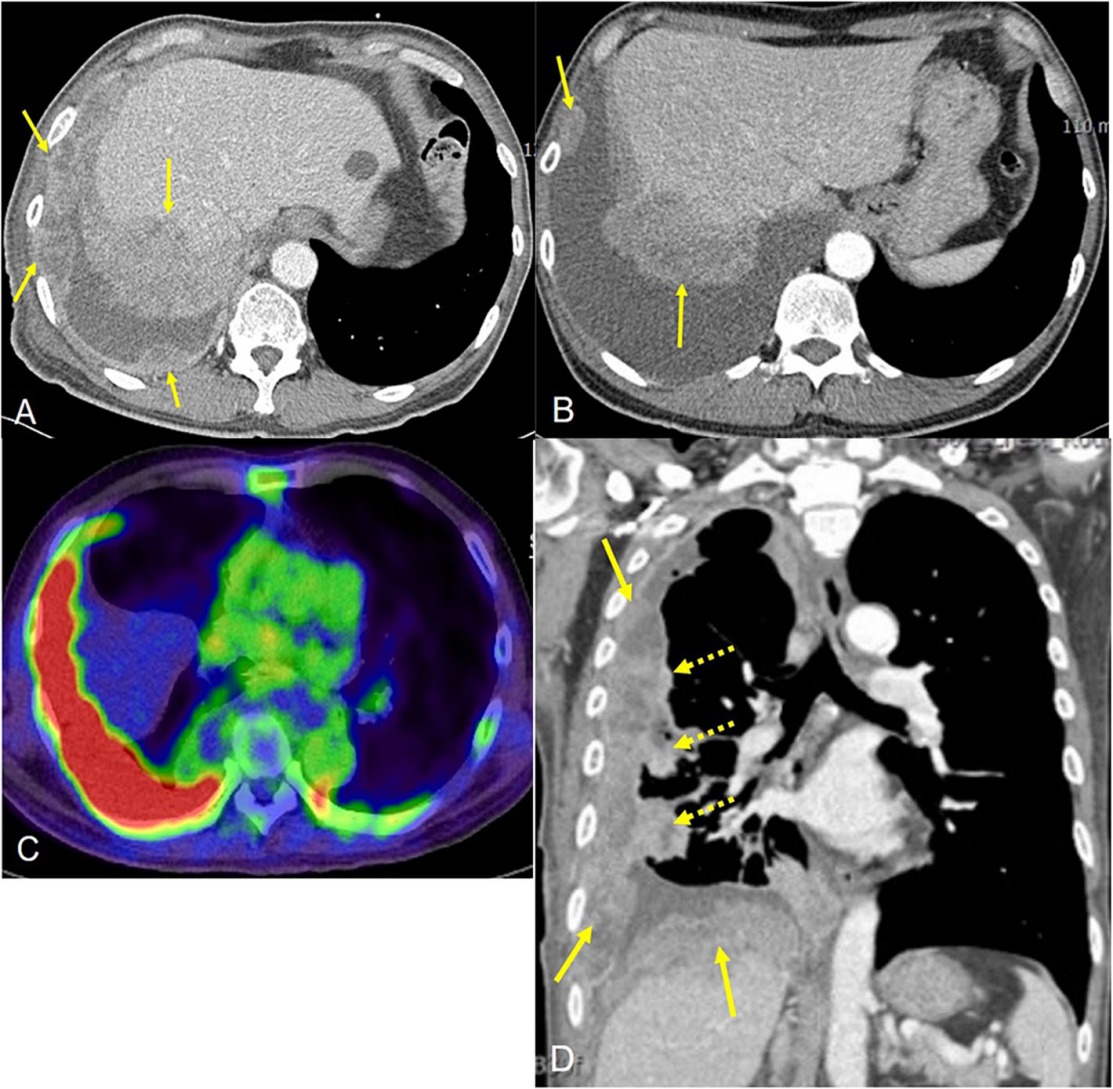
Enhanced multidetector CT (**A**, **B**) of a 67-year-old man showed right circumscribed, necrotic pleural masses. PET-CT(**C**) showed very intense uptake at the right pleural masses. 2D enhanced coronal reformat image (**D**) showed right visceral (dashed arrows) and parietal pleural masses(arrows)

**Fig. 4:**
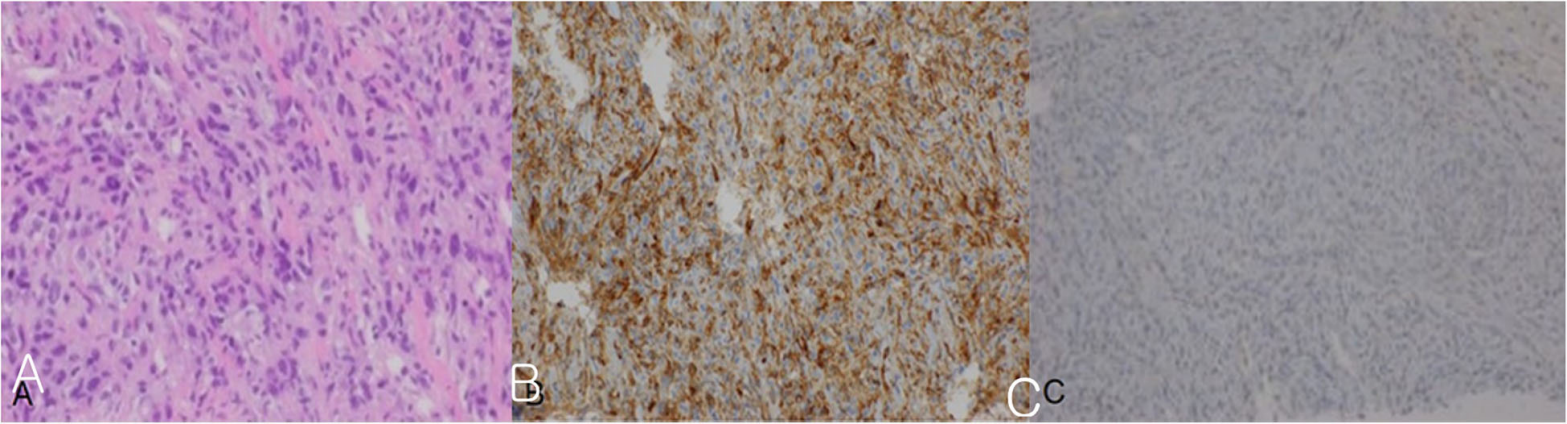
Pathology showed the tumor cells form solid sheet with multifocal luminal differentiation and vascular channel-like structures (**A**). Tumor cells show strong positive reaction for CD31 (**B**), supporting the diagnosis of vascular tumor. Tumor cells showed negative reaction for cytokeratin (**C**), revealing mesenchymal differentiation and extrapulmonary origin

The third patient was a 67-year-old male. He was a 40-pack-year current smoker, who visited our hospital for medical examination. Chest PA and chest CT showed right massive, circumscribed pleural masses and effusion (Fig. 3). These masses were conglomerated and necrotic. The pleural mass directly invaded the hepatic dome. PET-CT showed hot uptake on the right pleural masses. In addition, the sigmoid colon, right adrenal gland, right scapula, right 10^th^ and left 8^th^ ribs, and lumbar 4^th^ vertebral body and sacrum also showed FDG uptake. In a subsequent colonoscopy, moderately differentiated adenocarcinoma was diagnosed in polypectomy pathology. There was no marginal malignant involvement at that time. Thus, the likelihood of developing pleural metastasis from colon cancer was thought to be low. A right pleural biopsy and pleurodesis were performed. Surgery confirmed a right lower lobar mass, which was formed by the direct invasion of the pleural mass and about 4000 cc of hemorrhagic pleural fluid was evacuated. In immunohistochemistry, the tumors are all negative for carcinoma markers and TTF-1, and positive for vimentin, suggesting mesenchymal differentiation (Fig. 4). Also, CD31 was positively expressed and CK-7 and cytokeratin were negatively expressed and diagnosed as angiosarcoma. The patient was treated with chemotherapy (etoposide and cisplatin) for two cycles, but the malignant tumor continued to progress. The patient eventually expired four months after diagnosis.

We summarized 13 patients, including the given images of 10 PPA cases, reported in the English literature (Table 3). All of the 13 patients had pleural effusion except for two. Of the 11 patients, five showed bilateral pleural effusion and six showed unilateral pleural effusion. In the five patients with hemothorax, pleural nodules were not initially detected in three patients. Most of the masses were multiple and heterogenous (six of seven patients) on contrast-enhanced CT. Also, when examining the location of the pleural masses, there were parietal pleura in two cases, visceral and parietal pleura in three cases, and pleura and extrapleural masses in two cases.

**Table 3:** Summary of 13 patients (10 previously published cases with available CT images and our three cases)

Image findings	Number of patients
No pleural effusion	2
Pleural effusion(Bilateral/Unilateral)	11 (5/6)
High density effusion(Hemothorax) on CT	5 (2 hemothorax with nodule, 3 hemothorax without nodules)
Pleural nodule(Single/multiple)	7 (1/6)
Mass shape	Oval, irregular, lobulated
Mass location(A/B/C)*	(2/3/2)*
Heterogeneous / Homogeneous	Single (1), multiple (5)/multiple (1)
Mass enhancement on enhanced CT	6
Internal calcification	2 (1TB pleurisy sequela).
Directly invasion to adjacent structures	1 cardiac and chest wall invasion, 1 rib destruction, 1 liver invasion
Metastasis	1 gingival and skin; 1 multiple lung, rib, and axial skeleton metastasis

*A: parietal pleura, B: parietal and visceral pleura, C: pleural and extrapleural

*CT* Computed tomography, *TB* Tuberculosis

## Discussion

Angiosarcoma is a malignant vascular tumor, arising from endothelial cells. Primary pulmonary angiosarcoma is rare, with around 40 cases reported in the literature [3–6]. Among them, primary angiosarcoma originated from pleura or serous membrane is extremely rare, and when it occurs, it has an aggressive tendency. The histological epithelioid features of angiosarcoma, especially in pleural localization, gives rise to several differential diagnoses, including mesothelioma and metastatic epithelial cancers. The risk of being mistaken for epithelial tumor is high, because cytokeratine is expressed on this cases. Therefore, additional immunohistochemical studies play an important role in confirming that the tumor is of endothelial origin (5). Epithelioid angiosarcoma most often arises in the deep soft tissues (usually intramuscular) of the extremities, but a variety of primary sites, including the thyroid gland, skin, adrenal glands, and bone, have been encountered (9). It can also occur in the pleura, but the probability of occurrence is very low because it rarely received lymphovascular supply. Identifying areas showing vasoformative tendencies and immunostaining with endothelial markers, such as CD31, CD34, factor VIII, and FLI-1, are diagnostically important. Positive staining for CD31, CD34, and vimentin, as well as negative reactivity for epithelial markers (cytokeratin and CK7), together with morphologic criteria, led to the diagnosis of PPA (9).

In previously published articles, the authors emphasized the immunohistochemical differentiation between epithelioid angiosarcoma and endothelial malignancy of the pleura (2-8). In our study, immunohistochemical study is the gold standard for final diagnosis. However, since there were no previous reports based on MDCT scans, we decided to look at the recently discovered PPA cases from a radiological point of view. In our patients, all cases appeared as nodule forms, whereas the published reports showed pleural nodules in 40% of the patients (four of 10 cases). In the first patient, pleural nodules ranging from 1 – 2 cm were found in the parietal pleural layer with ipsilateral small pleural effusion on enhanced chest CT. In the contralateral ribcage, a large amount of pleural effusion was accompanied, but tiny parietal pleural nodules were few. We could think that angiosarcoma was developed at the site of the pleural nodule and then extended to the other side. Patient 3 presented with unilateral circumferential visceral and parietal necrotic masses about 2.8 – 10 cm and huge pleural effusion. It looked like malignant mesothelioma, but the patient did not have an occupational history. Although any highly enhancing areas were carefully searched, we did not see any foci as vascular-like enhancing structures or extravasation of the contrast media in the pleura. Instead we could recognize the hemorrhagic nature of the effusion because of the high-density pleural fluid on the pre-enhanced CT scan. Hemorrhagic pleural fluid (about 4000 cc) was evacuated in the surgical fields (pleurodesis). These two cases manifested as multiple pleural masses that could not be differentiated from metastatic pleural malignancies (lung, breast, ovary, stomach or lymphoproliferative origin) or mesothelioma on CT scans, or especially mesothelioma, even on pathology (9).

The last case, patient 2, showed huge pleural and extrapleural masses that were somewhat unique among the reported PPA cases. It is the only reported case of such a huge mass without pleural effusion. It seemed to be a kind of thoracic sarcoma (1). But, it had one characteristic; it was likely to be a pleural calcific sequela in the site of chronic TB pleurisy (arrows) (Fig. 2). Huge primary pleural tumors have been reported in chronic empyema sites. Any kind of sarcoma, such as fibro-, lipo-, rhabdomyo-, and leiomyosarcoma, mesothelioma and even, lymphoma can develop. The pathogenesis and etiology are still obscure. PPA has been described in the setting of chronic tuberculous pyothorax, foreign bodies, viral or other infection, empyema, asbestosis exposure, trauma (surgical excision), as well as a long-term consequence of radiotherapy, and even without any prior history as *de novo* tumors [3, 4, 7, 8].

This article has some limitations to report the cases. First of all, since the patients were collected retrospectively, it is not possible to add various trial. For example, chest MRI is the best tool to detect the chest wall tumor characteristics. Chest MRI can be a powerful tool to detect where the lesion originates such in nerve sheath tumors and also to evaluate tumor characteristics such as vascularity, necrosis, solid or cystic components and so on. However, we did not perform the chest MRI because of multicentered cases and cost problem. In the regard of imaging analysis, the three cases had varied imaging features that had little in common features suggesting the disease itself, except for pleural tumors. But, we would like to argue that this rare tumor appears diverse in the pleural and extrapleural spaces. It is difficult to diagnose with only imaging findings. We cannot predict primary pleural angiosarcoma with only these CT findings. Oure case series report focuses on pathologically confirming that the angiosarcomas in the pleural cavity is a primary type, not metastasis. We just introduce the variety of imaging features of pathologically proven primary pleural angiosarcoma

According to the reported literature, there are no specific imaging features that suggest PPA. In order to discriminate various diseases of the pleura, a history such as asbestosis exposure or other primary cancer occurrence will be the most import. However, it is difficult to differentiate pleura-origin mesothelioma or angiosarcoma based on radiological findings only in situations where clinical information is not clear. Both have localized mass and have nonspecific findings showing circumferential pleural masses, and aggressive proceeding along the surrounding pleura. It can be used to discriminate findings of pneumoconiosis in the lungs or with calcified pleural plaques. In this study, it was difficult to differntiate them, but we found a point that we should look carefully. We should be careful, even if it did not to look aggressive (benign-looking appearance). In the case of repeated large amounts of unilateral pleural effusion or hemothorax, the detection of tiny pleural nodules is important. The findings may also look similar to a hematoma [5]. If the extent of the lesion is small, and there is a trauma history, the diagnosis is very difficult. It is necessary to carefully examine the symptoms of the patient who does not improve or the lesion that does not decrease through continuous follow-up. Although pleural angiosarcoma is an extremely rare tumor, this dramatic clinical course should not be misdiagnosed, in order to give the patients the best chance for surgical or medical therapy.

The outlook for pleural angiosarcoma is dismal. Chemotherapy and surgery are the most common treatments. However, even chemotherapy has little effect, and is only used to relieve the symptoms. Even that can hardly be considered for patients with poor performance. The effect of radiation therapy is very insignificant and difficult to apply for distant metastasis or diffuse pleural metastasis. Surgery is the best treatment for those patients who have localized chest wall lesions and is the key to long-term survival. In addition to chemotherapy, surgery is known to help (surgical debulking, pneumonectomy, and so on) whenever possible [6, 9]. Vascular embolization can also be used to reduce the size of the tumors or reduce the complications during surgery. But most patients, like our patients, have a poor prognosis.

## Conclusion

Chest MDCT could detect more malignant pleural nodules in all of our PPA patients compared to the radiologic images of previous reports. Pleural nodules, especially circumferential visceral and parietal pleural nodules with or without localized extrapleural tumoral spread, were important radiologic findings of these extremely rare PPAs. Also, If hemorrhagic effusion was incidentally and repeatedly encountered without any other specific findings, we should consider rare disease.

## Supplementary Information

**Additional file 1.**

## Data Availability

Not applicable.

## Abbreviations

PPA: Primary pleural angiosarcoma
VATS: Video-assisted thoracic surgery
MDCT: Multidetector Computed Tomography
PET-CT: Positron emission tomography-computed tomography
FDG: Fluorodeoxyglucose
TB: Tuberculosis
CK: Cytokeratin
EMA: Epithelial membrane antigen
EA: Epithelioid angiosarcoma
R: Right
L: Left
