# Supplementary material for: Multidetector CT findings of primary pleural angiosarcoma : a systematic review, an additional cases report
Source: Cancer Imaging. 2022 Jan 12;22:6. doi: 10.1186/s40644-021-00435-1 (PMC8756718; doi:10.1186/s40644-021-00435-1)
Supplement: Supplementary file 1 — Additional file 1. [file 40644_2021_435_MOESM1_ESM.docx]

**Response to Reviewers**

Reviewer #1: Small case series. Although difficult to differentiate from imaging alone, need more insight into the disease. As mentioned in conclusion, we do not expect contrast extravasation unless there is a shunt or a fistula. Please consider doing radiomics on these cases.

- **I appreciate your comments. I agree that the article has some limitations to report about cases. The three cases had varied imaging features that had little in common features suggesting the disease itself, except for pleural tumors.**
- **But, we would like to argue that this rare tumor appears diverse in the pleural and extrapleural spaces. It is difficult to diagnose with only imaging findings. We can not predict primary pleural angiosarcoma with only these CT findings. Our case series report focuses on pathologically confirming that the angiosarcomas in the pleural cavity is a primary pleural angiosarcoma, not metastasis. We just introduce the variety of imaging features of pathologically proven primary pleural angiosarcoma.**
- **And I am sorry that this is a restrospective study. We will consider doing radiomics when we will meet the similar cases in some days as you recommend. We are going to delete the part of contrast extravasation in the text because we also can not make sure.**

Reviewer #2: please see my attached comments

In our patients, all cases appeared as nodule forms, whereas the published reports showed pleural nodules in 40% of the patients (four of 10 cases). This may be due to the improved image quality and the reduction in skip areas with thin slice scan intervals compared to past imaging. It may also be because the survival rate up to the time of mass formation has increased with improvements in medical technology. – This paragraph sounds very confusing, the prior reports didn’t show pleural nodules? Or your patient population had more pleural nodules compared to prior reports?, please explain better

- **This meaning of this paragraph is that pleural nodules were significantly better detected than in previous reports, because ours CTs were multidetectored CT applicating systems of thin collimation and rapid scan time. We have deleted some of the paragraphs**(tracted changes shown above, Page7 lines179-181) **pointed out by the reviewers.**

Is there any role for percutaneous biopsy? -- Please elaborate

- **CT guided biopsy using 17 gage(G) coaxial cutting needle played a major role to obtain not aspiration materials only but also the tissues to fix in the formalin. And these days it often replaces an open thoracoscopic biopsy. Specimens by CT guided biopy are mostly suitable for large extrapleural mass, such as in our cases. And these days it often replaces an open thoracoscopic biopsy.**

Does MRI has any role in elucidating where in the chest wall the lesion or tumor is arising from? Chest MR can be a powerful tool to detect where the lesion originates such in nerve sheath tumors and also to evaluate tumor characteristics such as vascularity, necrosis, solid vs cystic components, etc. Does any of your patients had chest MR? What does prior authors said about it.

- **I definitely agree with your opinion. Chest MRI is the best tool to detect the chest wall tumor characteristics. However, we did not perform the chest MRI in our cases because of multicentered cases and cost problem.**
- **“Chest MR can be a powerful tool to detect where the lesion originates such in nerve sheath tumors and also to evaluate tumor characteristics such as vascularity, necrosis, solid vs cystic components, etc.”**
- **We inserted your comment paragraph in our discussion**
